# Risk factors associated with suicide among leukemia patients: A Surveillance, Epidemiology, and End Results analysis

**DOI:** 10.1002/cam4.3502

**Published:** 2020-10-06

**Authors:** Haohui Yu, Ke Cai, Yulin Huang, Jun Lyu

**Affiliations:** ^1^ The First Affiliated Hospital of Jinan University Guangzhou China

**Keywords:** leukemia, risk factors, SEER, suicide

## Abstract

Previous studies have found that the risk of suicide is higher in patients diagnosed with cancer than in the general population. We aimed to identify potential risk factors associated with suicide in leukemia patients by analyzing data obtained from the Surveillance, Epidemiology, and End Results (SEER) database. We screened the SEER database for leukemia patients added between 1975 and 2017, and calculated their suicide rate and standardized mortality rate (SMR) relative to the total United States population from 1981 to 2017 as a reference. Univariate and multivariate Cox regression analyses were used to determine the risk factors for suicide in leukemia patients. We collected 142,386 leukemia patients who had been added to the SEER database from 1975 to 2017, of whom 191 patients committed suicide over an observation period of 95,397 person‐years. The suicide rate of leukemia patients was 26.41 per 100,000 person‐years, and hence the SMR of the suicided leukemia patients was 2.16 (95% confidence interval [CI] = 1.85–2.47). The univariate and multivariate Cox regression analyses showed that a high risk of suicide was associated with male sex (vs. female: hazard ratio [HR] = 4.41, 95% CI = 2.93–6.63, *p* < 0.001), older age at diagnosis (60–69 years vs. ≤39 years: HR = 2.60, 95% CI = 1.60–4.23, *p* < 0.001; 70–79 years vs. ≤39 years: HR = 2.84, 95% CI = 1.72–4.68, *p* < 0.001; ≥80 years vs. ≤39 years: HR = 2.94, 95% CI = 1.65–5.21, *p* < 0.001), white race (vs. black: HR = 6.80, 95% CI = 1.69–27.40, *p* = 0.007), acute myeloid leukemia (vs. lymphocytic leukemia: HR = 1.59, 95% CI = 1.09–2.33, *p* = 0.016), unspecified and other specified leukemia (vs. lymphocytic leukemia: HR = 2.72, 95% CI = 1.55–4.75, *p* < 0.001), and living in a small city (vs. large city: HR = 2.10, 95% CI = 1.23–3.60, *p* = 0.007). Meanwhile, being a non‐Hispanic black (vs. Hispanic: HR = 0.06, 95% CI = 0.01–0.62, *p* = 0.019) was a protective factor for suicide. Male sex, older age at diagnosis, white race, and acute myeloid leukemia were risk factors for suicide in leukemia patients, while being a non‐Hispanic black was a protective factor. Medical workers should, therefore, provide targeted preventive measures to leukemia patients with a high risk of suicide.

## Graphical abstract

We aimed to identify potential risk factors associated with suicide in leukemia patients by analyzing data obtained from the Surveillance, Epidemiology, and End Results (SEER) database. Male sex, older age at diagnosis, white race, and acute myeloid leukemia were risk factors for suicide in leukemia patients, while being a non‐Hispanic black was a protective factor. Medical workers should therefore provide targeted preventive measures to leukemia patients with a high risk of suicide.

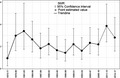

## 1 BACKGROUND

Suicide refers to an individual deliberately or voluntarily ending their own life under the action of complex psychological activities.^1^, ^2^, ^3^ Suicide is a complex global social phenomenon.^4^, ^5^ The World Health Organization reports that 817,000 people died of suicide globally in 2016, accounting for 1.49% of the total number of deaths, and corresponding to one person committing suicide every 40 s.^6^ The suicide mortality rate in the United States was 14.78 per 100,000 in 2018, which is relatively high compared with other countries.^7^

The prevalence of cancer has been increasing worldwide in recent years, and it is now the third leading cause of death.^8^ It was estimated that there were 18.1 million new cancer cases in 2018, with 9.6 million cancer deaths globally.^9^ Cancer treatments previously focused on prolonging life, while neglected the huge losses caused by cancer patients and their families during a long course of treatment and rehabilitation, such as the financial burden and mental illness.^10^, ^11^ The increasing incidence of cancer is also increasing the incidence of mental illness in patients during diagnosis and treatment, such as depression, fear of cancer recurrence, and suicidal thoughts.^9^, ^12^, ^13^ Several studies have found increases in suicidal ideation among cancer patients and that the suicide rate is higher in these patients.^2^, ^3^, ^4^, ^14^, ^15^, ^16^, ^17^, ^18^

The suicide rate in the United States is significantly higher in cancer patients than in the general population, with the suicide standardized mortality rate (SMR) reaching 4.44.^3^, ^19^ This situation indicates the importance of identifying the risk factors for suicide in cancer patients in order to be able to prevent their suicide behaviors. Some studies have found that male sex, white race, marital status, and other risk factors are strongly correlated with the suicide rate of patients with certain types of cancer, such as kidney cancer, breast cancer, lung cancer, and head and neck cancer.^13^, ^20^, ^21^, ^22^

Leukemia is a type of malignant proliferative disease involving hematopoietic stem cells.^23^ There were 437,033 new leukemia patients identified worldwide in 2018, accounting for 2.4% of all new cancer patients and ranking 15th among new cancer patients.^9^ Leukemia is a clinically common malignant tumor in the United States.^24^ Several studies have analyzed potential risk factors associated with suicide in common cancers in the United States using data obtained from the Surveillance, Epidemiology, and End Results (SEER) database.^13^, ^19^, ^21^, ^25^ However, no research into the relationship between leukemia and suicide has been reported in the literature. Therefore, the purpose of this study was to identify the potential risk factors related to leukemia and suicide by analyzing the SEER database.

## 2 METHODS

### 2.1 Data source

The SEER database of the National Cancer Institute covers 30% of the United States population and is considered to be representative of that population.^26^, ^27^ The SEER database provides registered researchers with free access to a large amount of research materials, including patient demographic, cancer incidence, and survival data. All of the leukemia patients selected for inclusion in this study had been added to the SEER database between 1975 and 2017. We obtained permission to access the database after signing and submitting the SEER Research Data Agreement form via email. The SEER*Stat software (version 8.3.6) was used to identify relevant patients for inclusion in this study.

### 2.2 Study population and inclusion criteria

**FIGURE 1:**
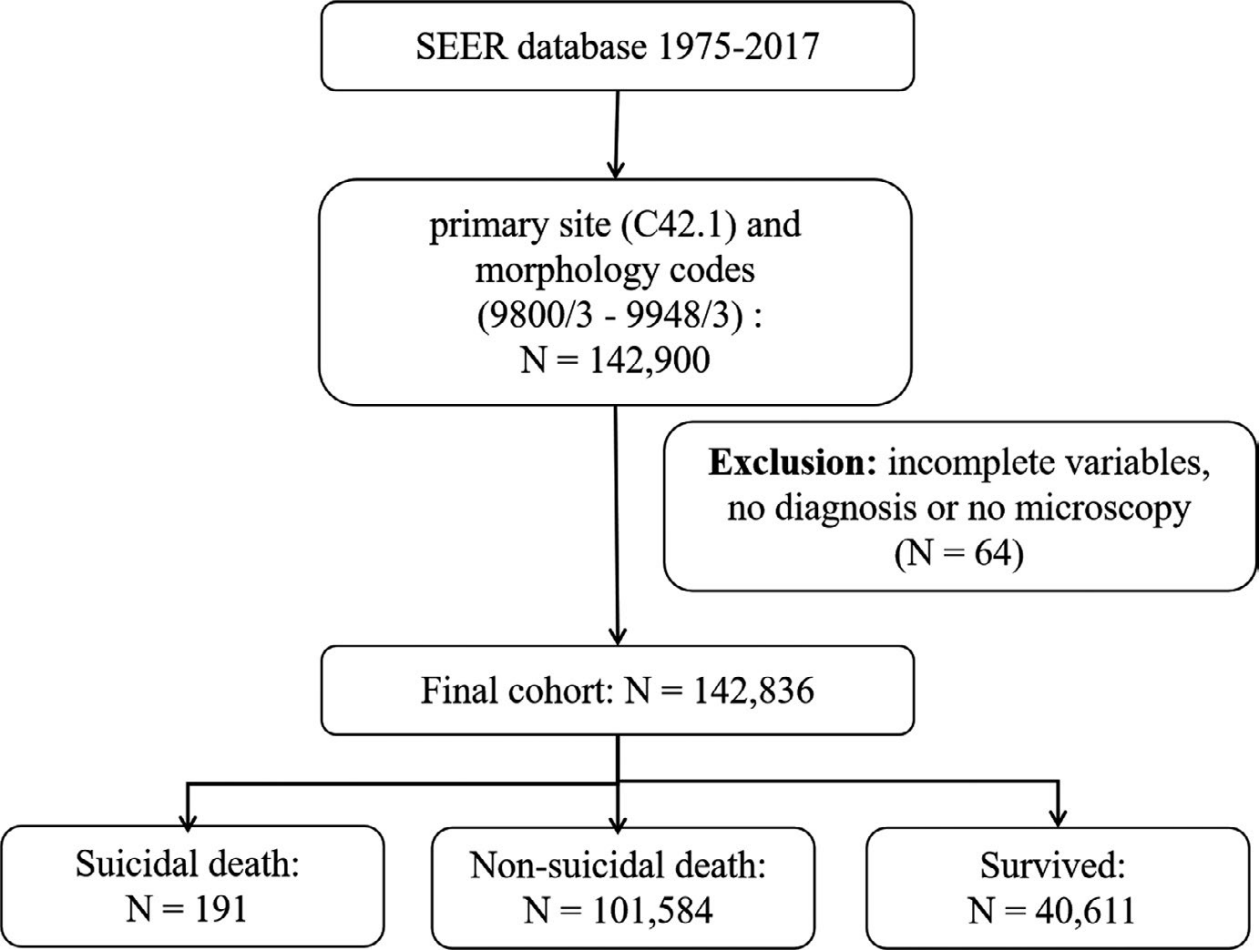
The flow diagram of leukemia patient selection

We collected all leukemia patients diagnosed during 1975–2017 as defined by International Classification of Disease for Oncology codes (third edition). Patients were identified using the primary site code (C42.1) and morphology codes (9800–9948) for leukemia. We considered that suicide had occurred in cases in which the cause of death was coded as “suicide and self‐mutilation.” The exclusion criteria included patients with incomplete variables and no diagnosis or microscopy findings. The information collected for all patients included the year of diagnosis, age at diagnosis, sex, race, type of leukemia, surgery status, survival period, and cause of death. The application of our selection criteria identified 142,386 leukemia patients who had been added to the SEER database from 1975 to 2017, of whom 191 had suicided. The steps used to select leukemia patients for inclusion in this study are shown in Figure 1.

### 2.3 Statistical analysis

We divided the patients into the following three groups to allow basic data comparisons: suicidal death, non‐suicidal death, and survival. The chi‐square test was used to compare suicide rates among patients in the different groups. We used the Web‐Based Injury Statistics Query and Reporting System of the United States Centers for Disease Control and Prevention (CDC)^7^ to calculate the SMR values for suicide in each group, with the general population of the United States from 1981 to 2018 used as the reference. The 95% confidence interval (CI) of the SMR was obtained using Byar's approximation.^28^ Subsequent univariate and multivariate Cox regression analyses were used to generate raw and adjusted hazard ratios (HR) and their associated 95% CI for identifying potential risk factors for suicide. All statistical analyses were performed using R software (version 3.6.3). All tests were double‐sided and had a significance criterion of *p* < 0.05.

## 3 RESULTS

### 3.1 Baseline characteristics of the patients

The 142,386 identified leukemia patients included 81,600 males (57.3%). While 191 patients died of suicide (0.13%), 101,574 patients died of other causes (71.34%) and 40,611 survived (28.53%). Most of the entire study population was older than 60 years (65.1%), white (86.3%), non‐Latin (94.4%), non‐Hispanic whites (81.1%), had lymphocytic leukemia (50.7%), and lived in a city (62.2%). The distribution was similar for the 191 suicided patients. The basic demographic data of each group of leukemia patients are presented in Table 1.

**TABLE 1:** Baseline characteristics of leukemia patients (1975–2017)

Variables	Overall N (%)	Suicidal death N (%)	Non‐suicidal death N (%)	Survived N (%)
Patients	142,386	191	101,584	40,611
Year of diagnosis				
1975–1984	23,382 (16.4%)	40 (21.0%)	22,110 (21.8%)	1232 (3.0%)
1985–1994	28,200 (19.8%)	44 (23.0%)	25,075 (24.7%)	3081 (7.6%)
1995–2004	34,324 (24.1%)	42 (22.0%)	26,657 (26.2%)	7625 (18.8%)
2005–2017	56,480 (39.7%)	65 (34.0%)	27,742 (27.3%)	28,673 (70.6%)
Sex				
Male	81,600 (57.3%)	164 (85.9%)	58,140 (57.2%)	23,296 (57.4%)
Female	60,786 (42.7%)	27 (14.1%)	43,444 (42.8%)	17,315 (42.6%)
Age at diagnosis				
≤39	22,098 (15.5%)	28 (14.7%)	9377 (9.2%)	12,693 (31.3%)
40–49	9287（6.5%)	13 (6.8%)	5208 (5.1%)	4066 (10.0%)
50–59	18,324 (12.9%)	21 (11.0%)	11,098 (10.9%)	7205 (17.7%)
60–69	29,198 (20.5%)	52 (27.2%)	20,675 (20.4%)	8471 (20.9%)
70–79	34,407 (24.2%)	48 (25.1%)	28,640 (28.2%)	5719 (14.1%)
≥80	29,072 (20.4%)	29 (15.2%)	26,586 (26.2%)	2457 (6.1%)
Race				
White	122,819 (86.3%)	185 (96.9%)	88,518 (87.1%)	34,116 (84.0%)
Black	10,402 (7.3%)	2 (1.0%)	7513 (7.4%)	2887 (7.1%)
Other	8263 (5.8%)	4 (2.1%)	5400 (5.3%)	2859 (7.0%)
Unknown	902 (0.6%)	0 (0.0%)	153 (0.2%)	749 (1.9%)
Race Latino				
Latino	7912 (5.6%)	13 (6.8%)	4471 (4.4%)	3428 (8.4%)
Non‐Latino	134,474 (94.4%)	178 (93.2%)	97,113 (95.6%)	37,183 (91.6%)
Race Hispanic				
Hispanic	7712 (5.4%)	13 (6.8%)	4471 (4.4%)	3428 (8.4%)
Non‐Hispanic White	115,506 (81.1%)	173 (90.6)	84,425 (83.1%)	30,908 (76.1%)
Non‐Hispanic Black	10,269 (7.2%)	2 (1.0%)	7444 (7.3%)	2823 (7.0%)
Non‐Hispanic Asian	7336 (5.2%)	3 (1.6%)	4857 (4.8%)	2476 (6.1%)
Non‐Hispanic American Indian Native	761 (0.5%)	0 (0.0%)	449 (0.4%)	312 (0.8%)
Non‐Hispanic unknown race	802 (0.6%)	0 (0.0%)	138 (0.1%)	664 (1.6%)
Type of leukemia				
Lymphoid leukemia	72,132 (50.7%)	111 (58.1%)	43,857 (43.2%)	28,164 (69.4%)
Acute myeloid leukemia	41,290 (29.0%)	38 (19.9%)	35,111 (34.6%)	6141 (15.1%)
Chronic myeloproliferative diseases	15,247 (10.7%)	22 (11.5%)	10,257 (10.1%)	4968 (12.2%)
Myelodysplastic syndrome and other myeloproliferative	3838 (2.7%)	3 (1.6%)	3274 (3.2%)	561 (1.4%)
Unspecified and other specified leukemia	9879 (6.9%)	17 (8.9%)	9085 (8.9%)	777 (1.9%)
Surgery performed				
Yes	81,180 (57.0%)	93 (48.7%)	46,384 (45.7%)	34,703 (85.5%)
No	61,206 (43.0%)	98 (51.3%)	55,200 (54.3%)	5908 (14.5%)
Primary diseases				
Yes	107,367 (75.4%)	147 (77.0%)	74,604 (73.4%)	32,616 (80.3%)
No	35,019 (24.6%)	44 (23.0%)	26,980 (26.6%)	7995 (19.7%)
Chemotherapy				
Yes	71,857 (50.5%)	91 (47.6%)	51,211 (50.4%)	20,555 (50.6%)
No	70,529 (49.5%)	100 (52.4%)	50,373 (49.6%)	20,056 (49.4%)
Household income				
<$50,000	10,412 (7.3%)	13 (6.8%)	6518 (6.4%)	3881 (9.6%)
$55,000–$74,999	52,348 (36.8%)	63 (33.0%)	33,197 (32.7%)	19,088 (47.0%)
>$75,000	42,529 (26.9%)	53 (27.7%)	27,378 (27.0%)	15,098 (37.2%)
Unknown	37,097 (26.0%)	62 (32.5%)	34,491 (33.9%)	2544 (6.2%)
Living area^a^				
Large city	58,952 (41.4%)	62 (32.5%)	36,985 (36.4%)	21,905 (53.9%)
Medium city	22,170 (15.6%)	25 (13.1%)	13,621 (13.4%)	8524 (21.0%)
Small city	7513 (5.3%)	17 (8.9%)	4801 (4.7%)	2695 (6.6%)
Suburbs	7960 (5.6%)	13 (6.8%)	5510 (5.4%)	2437 (6.0%)
Rural	7629 (5.4%)	10 (5.2%)	5296 (5.2%)	2323 (5.8%)
Unknown	38,162 (26.8%)	64 (33.5%)	35,371 (34.8%)	2727 (6.7%)

^a^ Large city, Counties in metropolitan areas of 1 million pop; Medium city, Counties in metropolitan areas of 250,000 to 1 million pop; Small city, Counties in metropolitan areas of lt 250 thousand pop; Suburbs, Nonmetropolitan counties adjacent to a metropolitan area; Rural, Nonmetropolitan counties not adjacent to a metropolitan area; Unknown, Unknown/missing/no match/Not 1990–2017.

### 3.2 Suicide rates and SMRs for the leukemia patients

The 142,386 leukemia patients included 191 patients who committed suicide over an observation period of 723,261 person‐years, giving a suicide rate of 26.41 per 100,000 person‐years. During the same period, the suicide rate in the general United States population was 12.24 per 100,000 person‐years,^7^ and so the SMR was 2.16 (95% CI = 1.85–2.47). The suicide rate was higher for the following characteristics than in the corresponding general United States population: male sex (SMR = 2.04: 95% CI = 1.75–2.39), older age at diagnosis (60–69 years: SMR = 2.40, 95% CI = 1.77–3.10; 70–79 years: SMR = 2.64, 95% CI = 1.97–3.54; ≥80 years: SMR = 2.99, 95% CI = 1.94–4.17), and white race (SMR = 2.11, 95% CI = 1.81–2.43). The suicide rate for the following characteristics did not differ from that in the corresponding general United States population: middle‐aged (40–49 years: SMR = 1.12, 95% CI = 0.58–1.85, *p* = 0.687; 50–59 years: SMR = 0.98, 95% CI = 0.62–1.53, *p* = 0.943), black race (SMR = 0.80, 95% CI = 0.11–3.61, *p* = 0.999), Asian race (SMR = 0.73, 95% CI = 0.73–2.19, *p* = 0.607), and living in a rural area (SMR = 2.38, 95% CI = 1.20–4.60, *p* = 0.054). The data for the suicide rate and SMR of the leukemia patients are presented in Table 2.

**TABLE 2:** Suicide rates and SMRs among patients with leukemia (1975–2017)

Variables	Suicidal death	Person‐years	Suicide rate per 100,000 person‐years		*p*	SMR^b^	95%CI
Patients	191	723,261	26.41		<0.001***	2.16	(1.85, 2.47)
Year of diagnosis							
1975–1984	40	132,341	30.22	<0.001***		2.47	(1.79, 3.4)
1985–1994	44	181,675	24.22	<0.001***		1.98	(1.45, 2.68)
1995–2004	42	216,971	19.36	0.003**		1.58	(1.12, 2.1)
2005–2017	65	192,274	33.81	<0.001***		2.76	(2.09, 3.45)
Sex							
Male	164	410,443	39.96	<0.001***		2.04	(1.75, 2.39)
Female	27	312,818	8.63	0.006**		1.68	(1.11, 2.46)
Age at diagnosis							
≤39	28	215,668	12.98	0.023*		1.53	(1.03, 2.25)
40–49	13	67,476	19.27	0.687		1.12	(0.58, 1.85)
50–59	21	118,604	17.71	0.943		0.98	(0.62, 1.53)
60–69	52	149,110	34.87	<0.001***		2.40	(1.77, 3.1)
70–79	48	119,131	40.29	<0.001***		2.64	(1.97, 3.54)
≥80	29	53,272	54.44	<0.001***		2.99	(1.94, 4.17)
Race							
White	185	632,876	29.23	<0.001***		2.11	(1.81, 2.43)
Black	2	45,831	4.36	0.999		0.80	(0.11, 3.61)
Other	4	39,266	10.19	0.455		1.45	(0.36, 3.41)
Unknown	0	5288	0.00	—		—	—
Race Latino							
Latino	13	42,402	30.66	0.001**		2.57	(1.38, 4.45)
Non‐Latino	178	680,859	26.14	<0.001***		2.19	(1.84, 2.48)
Race Hispanic							
Hispanic	13	42,402	30.66	<0.001***		2.57	(1.38, 4.45)
Non‐Hispanic white	173	592,237	29.21	<0.001***		2.45	(2.06, 2.79)
Non‐Hispanic Black	2	45,158	4.43	0.099		0.37	(0.04, 1.2)
Non‐Hispanic Asian	3	34,244	8.76	0.607		0.73	(0.15, 2.19)
Non‐Hispanic American Indian Native	0	4350	—	—		—	—
Non‐Hispanic Unknown Race	0	4871	—	—		—	—
Type of leukemia							
Lymphoid leukemia	111	529,480	20.96	<0.001***		1.76	(1.4, 2.06)
Acute myeloid leukemia	38	90,990	41.76	<0.001***		3.50	(2.44, 4.74)
Chronic myeloproliferative diseases	22	74,387	29.58	<0.001***		2.48	(1.53, 3.7)
Myelodysplastic syndrome and other myeloproliferative	3	7619	39.38	0.032*		3.30	(0.6, 8.77)
Unspecified and other specified leukemia	17	20,784	81.79	<0.001***		6.86	(3.3, 9.07)
Surgery performed							
Yes	93	346,125	26.87	<0.001***		2.25	(1.79, 2.71)
No	98	377,136	25.99	<0.001***		2.18	(1.73, 2.6)
Primary diseases							
Yes	147	549,586	26.75	<0.001***		2.24	(1.85, 2.58)
No	44	173,676	25.33	<0.001***		2.12	(1.52, 2.81)
Chemotherapy							
Yes	91	362,790	25.08	<0.001***		2.05	(1.63, 2.48)
No	100	360,472	27.74	<0.001***		2.27	(1.85, 2.76)
Household income							
<$50,000	13	40,995	31.71	<0.001***		2.59	(1.38, 4.45)
$55,000–$74,999	63	246,589	25.55	<0.001***		2.09	(1.61, 2.69)
>$75,000	53	216,490	24.48	<0.001***		2.00	(1.52, 2.67)
Unknown	62	219,187	28.29	<0.001***		2.31	(1.43, 2.53)
Living area^a^							
Large city	62	284,755	21.77	<0.001***		1.83	(1.36, 2.27)
Medium city	25	103,006	24.27	<0.001***		2.03	(1.24, 2.84)
Small city	17	37,331	45.54	<0.001***		3.82	(1.98, 5.44)
Suburbs	13	37,094	35.05	0.003**		2.94	(1.38, 4.45)
Rural	10	35,260	28.36	0.054		2.38	(1.2, 4.6)
Unknown	64	225,816	28.34	<0.001***		2.38	(1.76, 2.92)

a Large city, Counties in metropolitan areas of 1 million pop; Medium city, Counties in metropolitan areas of 250,000 to 1 million pop; Small city, Counties in metropolitan areas of lt 250 thousand pop; Suburbs, Nonmetropolitan counties adjacent to a metropolitan area; Rural, Nonmetropolitan counties not adjacent to a metropolitan area; Unknown, Unknown/missing/no match/Not 1990–2017.

^b^ SMR, standardized mortality ratio: Compared with the suicide rates of the general US population based on the Centers for Disease Control and Prevention's Web‐based Injury Statistics Query and Reporting System (1981–2017).

* *p* < 0.05.

** *p* < 0.01.

*** *p* < 0.001.

**FIGURE 2:**
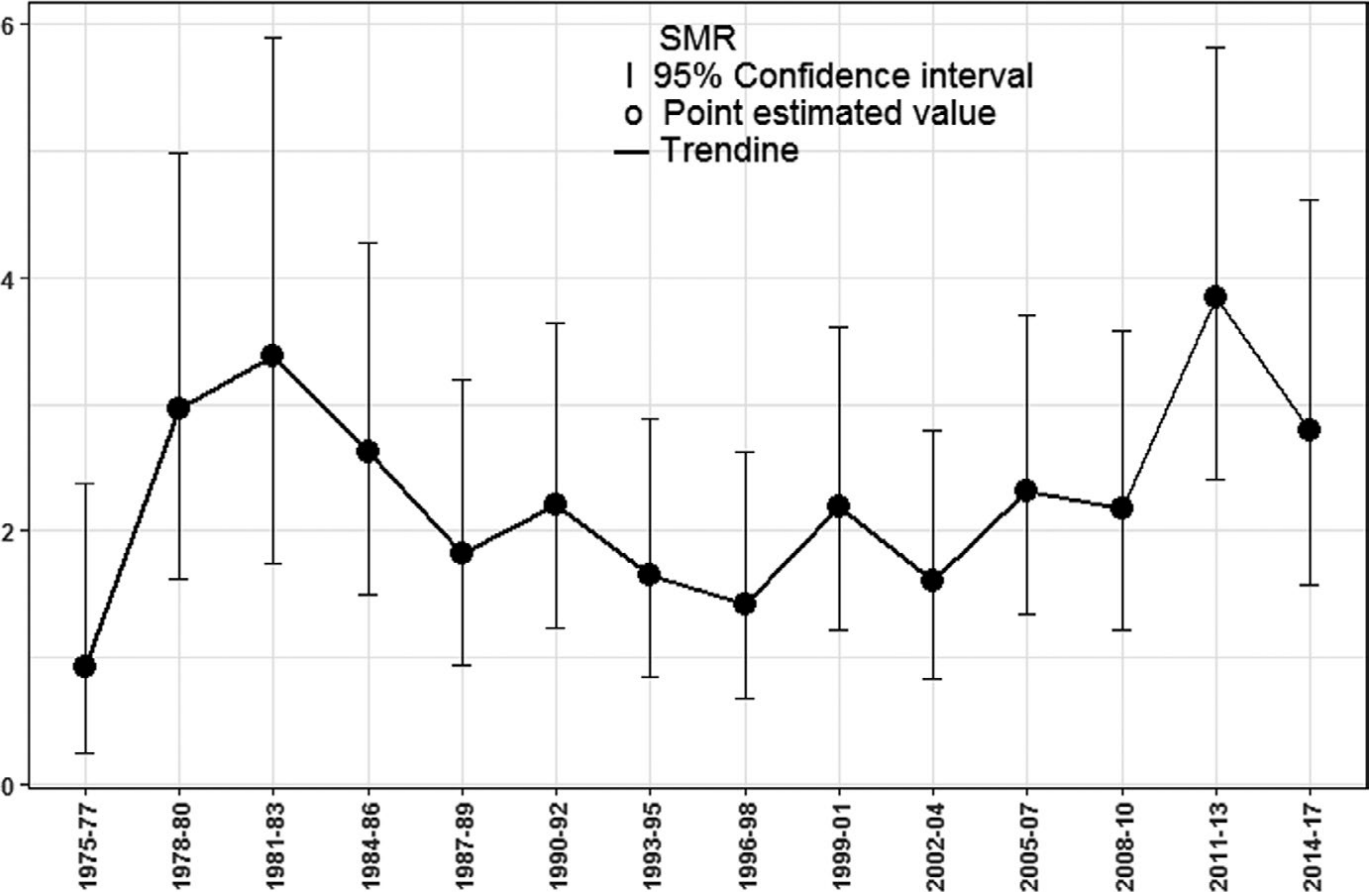
Standardized mortality ratio of suicide for leukemia patients (1975–2017)

Since the CDC does not have data on the suicide mortality rate of the general population from 1975 to 1980, the population suicide benchmark from 1981 to 1983 was used in this study to adjust the suicide rate of leukemia patients from 1975 to 1980.^7^ It was found that between 1975 and 2017, the SMR for suicide in leukemia patients in the United States fluctuated between 1.0 and 4.0. The changes in SMR of suicide in leukemia patients are shown in Figure 2.

### 3.3 Factors associated with suicide

**FIGURE 3:**
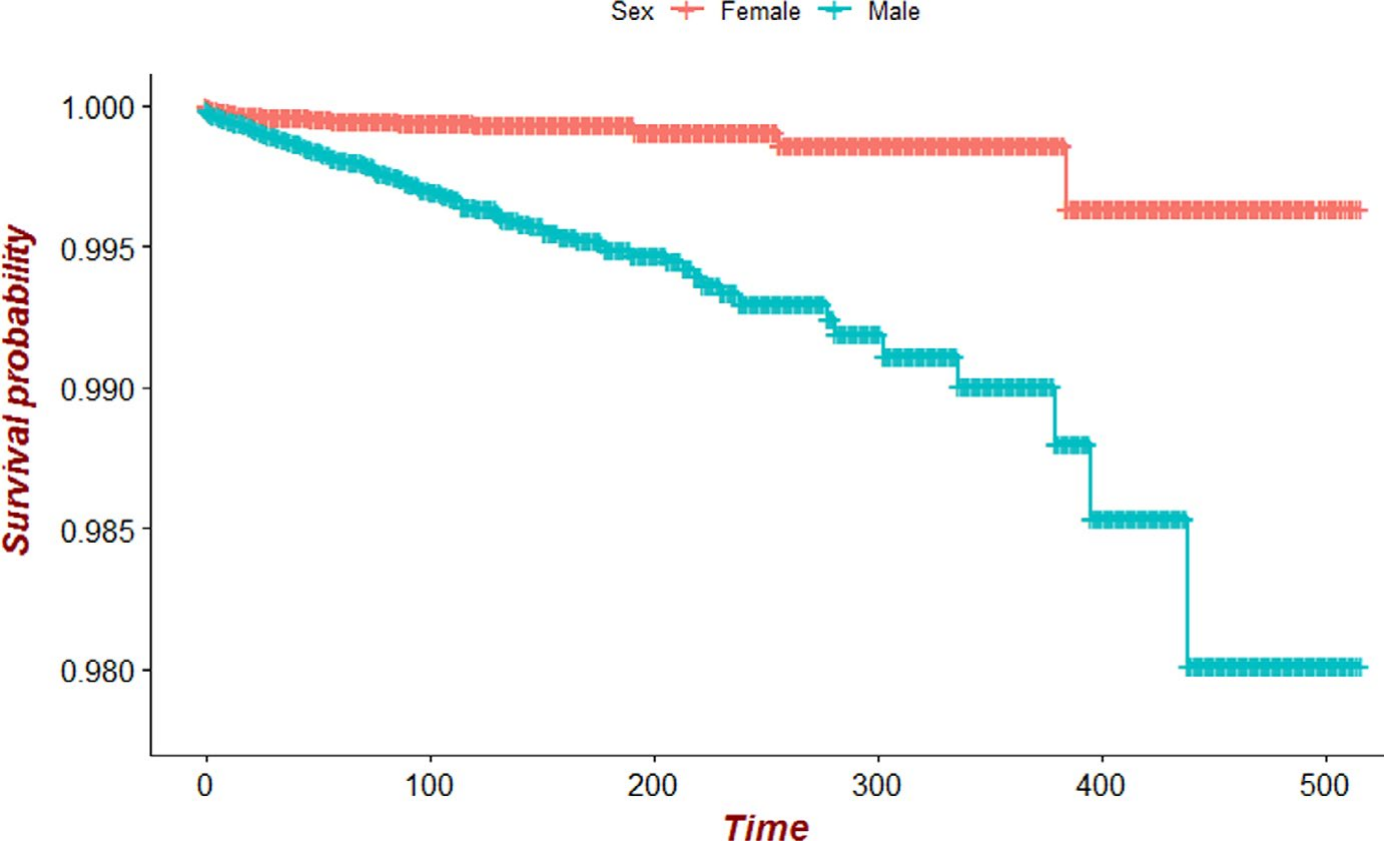
Comparison chart of survival curves of male and female leukemia patients

The univariate analysis showed that the factors associated with a higher risk of suicide were male sex (vs. female: HR = 4.41, 95% CI = 2.93–6.63, *p* < 0.001), older age at diagnosis (60–69 years vs. ≤39 years: HR = 2.60, 95% CI = 1.60–4.23, *p* < 0.001; 70–79 years vs. ≤39 years: HR = 2.84, 95% CI = 1.72–4.68, *p* < 0.001; ≥80 years vs. ≤39 years: HR = 2.94, 95% CI = 1.65–5.21, *p* < 0.001), white race (vs. black: HR = 6.80, 95% CI = 1.69–27.40, *p* = 0.007), leukemia type (acute myeloid leukemia vs. lymphocytic leukemia: HR = 1.59, 95% CI = 1.09–2.33, *p* = 0.016; unspecified and other specified leukemia vs. lymphocytic leukemia: HR = 2.72, 95% CI = 1.55–4.75, *p* < 0.001), and living in a small city (vs. large city: HR = 2.10, 95% CI = 1.23–3.60, *p* = 0.007). Meanwhile, being a non‐Hispanic black (vs. Hispanic: HR = 0.15, 95% CI = 0.03–0.66, *p* = 0.013) was associated with a lower risk of suicide. The multivariate Cox regression analysis showed that the factors associated with a higher risk of suicide were male sex (vs. female: HR = 4.73, 95% CI = 3.13–7.11, *p* < 0.001), older age at diagnosis (60–69 year vs. ≤39 years: HR = 2.89, 95% CI = 1.73–4.81, *p* < 0.001; 70–79 years vs. ≤39 years: HR = 3.36, 95% CI = 1.98–5.69, *p* < 0.001; ≥80 years vs. ≤39 years: HR = 3.85, 95% CI = 2.10–7.04, *p* < 0.001), white race (vs. black: HR = 5.90, 95% CI = 1.46–23.84, *p* = 0.013), leukemia type (acute myeloid leukemia vs. lymphocytic leukemia: HR = 2.27, 95% CI = 1.53–3.37, *p* < 0.001; unspecified and other specified leukemia vs. lymphocytic leukemia: HR = 3.21, 95% CI = 1.82–5.66, *p* < 0.001), and living in a small city (vs. large city: HR = 2.16, 95% CI = 1.18–3.95, *p* = 0.012). Meanwhile, being non‐Hispanic (non‐Hispanic black vs. Hispanic: HR = 0.06, 95% CI = 0.01–0.62, *p* = 0.019; non‐Hispanic Asian vs. Hispanic: HR = 0.23, 95% CI = 0.13–0.85, *p* = 0.032) was associated with a lower risk of suicide. The risk factors related to suicide in leukemia patients are presented in Table 3. A subsequent Cox survival regression analysis revealed that the risk of suicide was higher in male leukemia patients than in female patients (Figure 3).

**TABLE 3:** Univariable and multivariable analysis for suicide of leukemia patients

Variables	Univariable analysis		Multivariable analysis	
	HR(95%CI)	*p*	HR(95%CI)	*p*
Year of diagnosis				
1975–1984	Reference		Reference	
1985–1994	0.89 (0.57–1.40)	0.616	0.87 (0.50–1.51)	0.619
1995–2004	0.72 (0.46–1.14)	0.165	0.53 (0.21–1.38)	0.192
2005–2017	0.99 (0.64–1.53)	0.977	0.70 (0.25–1.97)	0.493
Sex				
Female	Reference		Reference	
Male	4.41 (2.93–6.63)	<0.001***	4.73 (3.13–7.11)	<0.001***
Age at diagnosis				
≤39	Reference		Reference	
40–49	1.41 (0.71–2.79)	0.33	1.35 (0.68–2.70)	0.391
50–59	1.32 (0.73–2.38)	0.356	1.37 (0.75–2.50)	0.303
60–69	2.60 (1.60–4.23)	<0.001***	2.89 (1.73–4.81)	<0.001***
70–79	2.84 (1.72–4.68)	<0.001***	3.36 (1.98–5.69)	<0.001***
≥80	2.94 (1.65–5.21)	<0.001***	3.85 (2.10–7.04)	<0.001***
Race				
Black	Reference		Reference	
White	6.80 (1.69–27.40)	0.007**	5.90 (1.46–23.84)	0.013*
Other	2.43 (0.45–13.28)	0.305	2.46 (0.45–13.48)	0.298
Unknown	—	—	—	—
Race Latino				
Latino	Reference		Reference	
Non‐Latino	1.12 (0.62–2.01)	0.702	1.22 (0.65–2.28)	0.530
Race Hispanic				
Hispanic	Reference		Reference	
Non‐Hispanic White	1.00 (0.56–1.79)	0.998	0.80 (0.43–1.48)）	0.993
Non‐Hispanic Black	0.15 (0.03–0.66)	0.013*	0.06（0.01–0.62）	0.019*
Non‐Hispanic Asian	0.31 (0.09–1.08)	0.067	0.23（0.13–0.85）	0.032*
Non‐Hispanic American Indian Native	—	—	—	—
Non‐Hispanic Unknown Race	—	—	—	—
Type of leukemia				
Lymphoid leukemia	Reference		Reference	
Acute myeloid leukemia	1.59 (1.09–2.33)	0.016*	2.27 (1.53–3.37)	<0.001***
Chronic myeloproliferative diseases	1.24 (0.78–1.98)	0.366	1.60 (0.99–2.56)	0.052
Myelodysplastic syndrome and other myeloproliferative	1.32 (0.42–4.12)	0.634	1.13 (0.35–3.59)	0.838
Unspecified and other specified leukemia	2.72 (1.55–4.75)	<0.001***	3.21 (1.82–5.66)	<0.001***
Surgery performed				
Yes	1.05 (0.78–1.42)	0.748	1.43 (0.68–3.02)	0.339
No	Reference		Reference	
Primary diseases				
Yes	1.03 (0.73–1.44)	0.88	1.34 (0.95–1.90)	0.098
No	Reference		Reference	
Chemotherapy				
Yes	0.862 (0.65–1.15)	0.312	0.88 (0.66–1.17)	0.370
No	Reference		Reference	
Household income				
<$50,000	Reference		Reference	
$55,000–$74,999	1.00 (0.69–1.45)	0.996	1.27 (0.83–1.95)	0.279
>$75,000	1.19 (0.66–2.17)	0.564	1.04 (0.54–2.00)	0.904
Unknown	1.12 (1.77–1.63)	0.548	0.51 (0.11–2.46)	0.402
Living area^a^				
Large city	Reference		Reference	
Medium city	1.06 (0.66–1.69)	0.821	1.03 (0.63–1.68)	0.903
Small city	2.10 (1.23–3.60)	0.007**	2.16 (1.18–3.95)	0.013*
Suburbs	1.59 (0.88–2.90)	0.127	1.48 (0.78–2.83)	0.234
Rural	1.29 (0.66–2.52)	0.455	1.23 (0.58–2.65)	0.592
Unknown	1.29 (0.77–1.87)	0.174	2.51 (0.57–10.96)	0.223

^a^ Large city, Counties in metropolitan areas of 1 million pop; Medium city, Counties in metropolitan areas of 250,000 to 1 million pop; Small city, Counties in metropolitan areas of lt 250 thousand pop; Suburbs, Nonmetropolitan counties adjacent to a metropolitan area; Rural, Nonmetropolitan counties not adjacent to a metropolitan area; Unknown, Unknown/missing/no match/Not 1990–2017.

* *p* < 0.05.

** *p* < 0.01.

*** *p* < 0.001.

## 4 DISCUSSION

Multiple studies have confirmed that the suicide mortality rate is higher in cancer patients than in the general population.^3^, ^4^, ^17^ Innos K reported that the suicide rate in Estonian cancer patients was higher than that in the general population,^29^ while Crocetti E found the same result for Italian cancer patients.^16^ The suicide rate is about twofold higher in cancer patients than the general population in the United States.^19^ Using the SEER database, we found that the suicide rate of leukemia patients in the United States from 1975 to 2017 was approximately 26.41 per 100,000 person‐years, compared to 12.24 per 100,000 person‐years in the general population, giving an SMR of 2.16 (95% CI = 1.85–2.47), which is similar to the result that other cancer patients have a higher suicide rate than the general population.^19^ The important risk factors for suicide in leukemia patients include male sex, older age at diagnosis, white race, and acute myeloid leukemia.

We found that the proportion of male leukemia patients in the United States is similar to that of females (Table 1), whereas most suicided leukemia patients are males (85.9%), with a suicide rate of 39.96 per 100,000 person‐years, compared to 8.63 per 100,000 person‐years in female leukemia patients (*p* < 0.001) (Table 2). In addition, male leukemia patients in the United States are more likely to commit suicide than are than female patients, with an HR of 4.41 (Table 3), which is similar to the suicide rates for male patients with other cancers.^19^, ^30^ Despite the similar prevalence rates of male and female leukemia patients, the much higher suicide rate in male leukemia patients may be related to the poor ability of males to withstand psychological pressures and their tendency for self‐directed violence.^31^, ^32^

We found that most of the leukemia patients were older than 60 years (65.1%), and the suicide rate was higher in this age group (60–69 years vs. ≤39 years: HR = 2.60, 95% CI = 1.60–4.23, *p* < 0.001; 70–79 years vs. ≤39 years: HR = 2.84, 95% CI = 1.72–4.68, *p* < 0.001; ≥80 years vs. ≤39 years: HR = 2.94, 95% CI = 1.65–5.21, *p* < 0.001) (Table 3). In general, the incidence rates of leukemia and suicide increased with the age of the patients, which is consistent with previous studies of elderly patients having other types of cancer.^22^, ^33^, ^34^ The higher suicide rates of elderly leukemia patients might be related to their quality of life, outlook about life and death, and psychological condition such as the presence of depression.^11^, ^35^, ^36^

Previous research has shown that the suicide rate among cancer patients in the United States is highest for whites and lowest for blacks.^37^, ^38^ Our results similarly found that among all racial groups in the United States, the risk of suicide was on average 6.80‐fold higher for whites than for blacks (95% CI = 1.69–27.40, *p* = 0.007) (Table 3). Compared with Hispanics, the suicide rates of non‐Hispanic blacks and non‐Hispanic Asians were 15% and 31% lower, respectively (Table 3). These results indicate that being white is a risk factor for suicide, while being black is a protective factor, which might be related to differences in the level of knowledge and culture among racial groups, religious beliefs, and economic conditions.^12^, ^36^, ^39^, ^40^

We also found that acute myeloid leukemia and unspecified and other specified leukemia were high‐risk factors for suicide in leukemia patients. In the SEER database, most of the leukemia patients in the United States have lymphocytic leukemia (50.7%) who also account for the majority of suicided patients (58.1%), and their SMR is also higher than in the general population (SMR = 1.76, 95% CI = 1.40–2.06) (Tables 1 and 2). However, compared with lymphoid leukemia, patients with acute myeloid leukemia had a 2.27‐fold higher risk of suicide (95% CI = 1.53–3.37, *p* < 0.001), while among those with unspecified and other specified leukemia it was 3.21‐fold higher (95% CI = 1.82–5.66, *p* < 0.001) (Table 3). The prognosis varies with the type of cancer, and this often causes changes in many characteristics of affected patients, such as physical status, psychological status, and quality of life, which in turn lead to an increased risk of suicide.^19^, ^36^ Acute myelogenous leukemia often occurs in young adults, and the disease progresses rapidly,^41^ with some patients dying within a few months after the disease onset.^42^ The unspecified and other specified leukemia have unclear disease types, poor treatment effects, and short survival times.^43^ The low remission rate for these two types of leukemia patients exerts huge economic pressures and psychological burdens on them. Resulting factors such as discrimination, inferiority, and depression have led to their high suicide rate compared with other types of leukemia patients.^44^

Finally, as shown in Figure 1, we found that the SMR for suicide among leukemia patients peaked in the 1980s and 2010s, and reached a nadir during 1975–1977 (SMR = 0.93, 95% CI = 0.25–2.37). Because the CDC lacks data on suicide mortality for the general population before 1980, the suicide rate of leukemia patients from 1975 to 1980 was adjusted to the population suicide benchmark from 1981 to 1983,^7^ which may have reduced the adjusted SMRs. Although the SMR of leukemia patients generally fluctuated between 1.5 and 3.0 after the 1980s, it peaked in the 2010s at 3.84 (95% CI = 2.41–5.82). This may be related to factors such as financial crises, high medical costs, declining quality of life, and psychological burden.^45^, ^46^

Previous research has confirmed that some interventions can help to prevent suicide,^5^ and that the suicide behavior of cancer patients is affected by various factors such as the psychological status, economic status, and religious beliefs.^34^, ^45^ Psychological factors such as depression are especially important risk factors for suicide.^12^ Our research results indicated that the suicide rate of leukemia patients is higher than that of the general population. In order to reduce suicide in leukemia patients, psychiatric evaluations should be applied to this population. Some tools are available for identifying the risk of depression, including the Baker Depression Scale.^31^ Providing psychological treatment to patients with leukemia at risk of depression can help to reduce the risk of suicide, as can the application of active treatment, improving their quality of life, and strengthening communication between family members.^47^

Our research was subject to some limitations. We used SEER*Stat software (version 8.3.6) to collect patient information, but some information is currently missing from the system, such as the marital status, medication status, chemotherapy status, social factors, and psychological factors. These factors may impact suicide, and so the lacking data might have biased the risk assessments of suicide factors in leukemia patients. In this study, we collected leukemia patients from 1975 to 2017 who may have affected their health or even committed suicide due to the psychiatric illness. The SEER database is unable to obtain information on the diagnosis of leukemia patients with psychiatric, which affects the results of the study. Moreover, the SEER database only collects data from a proportion of cancer patients in the United States. Therefore, the suicide risk of leukemia patients should also be assessed in other countries.

## 5 CONCLUSIONS

We have investigated the risk of suicide in leukemia patients and the underlying independent risk factors. Male sex, older age at diagnosis, white race, and acute myeloid leukemia were found to be significant risk factors for suicide in leukemia patients, while being a non‐Hispanic black was a protective factor. Medical workers can use our research results to screen leukemia patients with a higher risk of suicide and apply targeted preventive measures to them.

## CONFLICT OF INTEREST

None.

## AUTHOR CONTRIBUTIONS

Haohui Yu and Yulin Huang performed statistical analysis and data interpretation. Haohui Yu, Ke Cai, and Jun Lyu contributed to the study concept and study design. Haohui Yu and Yulin Huang performed literature research and data extraction. Ke Cai and Jun Lyu were responsible for the quality control of data and algorithms. All authors contributed to writing of the manuscript and approved the final version.

## Data Availability

We obtained permission to access the database after signing and submitting the SEER Research Data Agreement form via email. We used SEER*Stat software (version 8.3.6) to identify US leukemia patients who were added to the SEER database (http://seer.cancer.gov). All statistical analyses were performed using R software (version 3.6.3, http://www.r‐project.org/).
